# Routinely Measured Hematological Markers Can Help to Predict American Spinal Injury Association Impairment Scale Scores after Spinal Cord Injury

**DOI:** 10.1089/neu.2020.7144

**Published:** 2021-01-15

**Authors:** Gabriel Mateus Bernardo Harrington, Paul Cool, Charlotte Hulme, Aheed Osman, Joy Roy Chowdhury, Naveen Kumar, Srinivasa Budithi, Karina Wright

**Affiliations:** ^1^ Keele University, Staffordshire, United Kingdom.; ^2^ Robert Jones and Agnes Hunt Orthopedic Hospital NHS Foundation Trust, Oswestry, United Kingdom.

**Keywords:** biomarker, blood, modeling, neurology, spinal cord injury

## Abstract

Neurological outcomes following spinal cord injury (SCI) are currently difficult to predict. While the initial American Spinal Injury Association Impairment Scale (AIS) grade can give an estimate of outcome, the high remaining degree of uncertainty has stoked recent interest in biomarkers for SCI. This study aimed to assess the prognostic value of routinely measured blood biomarkers by developing prognostic models of AIS scores at discharge and 12 months post-injury. Routine blood and clinical data were collected from SCI patients (*n* = 417), and blood measures that had been assessed in less than 50% of patients were excluded. Outcome neurology was obtained from AIS and Spinal Cord Independence Measure III (SCIM-III) scores at discharge and 12 months post-injury, with motor (AIS) and sensory (AIS, touch and prick) abilities being assessed individually. Linear regression models with and without elastic net penalization were created for all outcome measures. Blood measures associated with liver function, such as alanine transaminase, were found to add value to predictions of SCIM-III at discharge and 12 months post-injury. Further, components of a total blood count, including hemoglobin, were found to add value to predictions of AIS motor and sensory scores at discharge and 12 months post-injury. These findings corroborate the results of our previous preliminary study and thus provide further evidence that routine blood measures can add prognostic value in SCI and that markers of liver function are of particular interest.

## Introduction

Spinal cord injury (SCI) is damage to the spinal cord due to trauma, degeneration, or disease that results in a temporary or permanent change to its neurological function. The global age-standardized incidence of SCI has been estimated to be 13 per 100,000, whereas the age-standardized prevalence was estimated to be 368 per 100,000.^1^ With respect to the United Kingdom, it has been estimated that over 1000 new SCIs occur each year, and that 40,000 people are living with SCI.^2^ The majority of SCIs have historically been traumatic in nature, most commonly as a result of vehicular accidents, falls, violence, and sports. But more recently, non-traumatic SCI, usually as a result of infection or cancer, has been increasing in prevalence.^3,4^

The lifetime cost of SCI in the UK is estimated to be £1.12 million (mean value) per case, with the total cost of SCI in 2016 in the UK being £1.43 billion.^5^ SCI can lead to secondary conditions that increase morbidity and mortality, including respiratory complications, deep vein thrombosis, muscle spasms, urinary tract infections, osteoporosis, pressure ulcers, risk of fracture, and chronic pain. Further, patients with SCI are often rendered dependent on caregivers and show markedly higher rates of mental illness relative to the general population.^6^

There is a challenge in the development of novel therapeutic interventions for SCI, with only four large-scale clinical trials having been tested in acute SCI, three of which evaluated methylprednisolone and one evaluated GM-1 ganglioside.^7–10^ This is due to the SCI population being inherently heterogeneous and experiencing a highly variable degree of “natural” recovery.^11^ Currently, the best predictor of neurological outcome is the initial measure of neurologic impairment, as assessed with the International Standards for Neurological Classification of SCI (ISNCSCI) examination.^12^ However, the ISNCSCI examination was not intended to be predictive of functional recovery, and it has been found that changes in American Spinal Injury Association Impairment Scale (AIS) grade do not necessarily indicate meaningful changes to daily living for patients.^13^ Robust SCI biomarkers could help stratify patients such that their baseline functional recovery could be predicted, allowing any potential novel therapies to be properly assessed, thus accelerating research and clinical trials in particular via covariate adjustment.^14^

A reliable prognostic model of SCI would also allow healthcare providers to better plan patient care, relieve patients of potentially damaging psychological uncertainty, and could highlight new avenues of research.^15^ While relatively few studies have sought to identify prognostic biomarkers for SCI, recent years have seen some early/discovery phase publications.^16–19^ These preliminary studies have largely focused on biomarkers in cerebral spinal fluid during the acute phase of injury, with little information regarding the chronic or recovery phase. Even among these studies, however, there has been little investigation as to the value of blood biomarkers in SCI at any injury phase, despite success in other fields, including cancer, traumatic brain injury, and Alzheimer's disease.^20-22^

We previously published a preliminary study that highlighted the value of routinely measured blood analytes in prognostic models of SCI, and demonstrated that some blood measures, particularly markers of liver function, added modest but statistically significant value to predictions of 3- and 12-month ISNSCI AIS motor and sensory scores.^23^ In this study, we have validated our findings in another, independent and larger SCI cohort. We have further developed alternative, more robust methods of modeling and have demonstrated that similar markers, including alanine transaminase (ALT) and gamma-glutamyl transferase (GGT) add value not only when predicting AIS scores at discharge and 12 months, but also with regard to Spinal Cord Independence Measure (SCIM) outcomes.

## Methods

### Patient and model feature summary

We retrospectively studied the electronic health records of 500 patients who had been admitted to the Midlands Centre for Spinal Injuries in the last 10 years (Table 1). Access to these records was ethically approved by the National Research Ethics Service (NRES) Committee North West Liverpool East (11/NW/0876) and NRES Committee West Midlands, Staffordshire (13/WM/0158). Following the exclusion of patients who had been admitted over 6 months post-injury, 73 individuals were removed from further analysis.

**Table 1.** Patient Demographics

		Number of SCI patients (*n *out of 417)	Percent
	Age at injury (median years)	56 ± 28	
	Length of stay (median days)	100 ± 66	
	Fracture	225	53
	Surgery	217	51
	Traumatic injury	319	75
	Type 1 diabetes	5	1
Smoker	Type 2 diabetes	44	10
	No	281	66
	Yes	52	12
Alcohol consumption	Unknown	84	20
	No	181	42
	Yes	152	36
Gender	Unknown	84	20
	Male	283	66
Time from injury (median days)	Female	134	31
	First blood test	22 ± 35	
	Admission	20 ± 34	
	Discharge	128 ± 82	
Neurological level of injury	Month-12 assessment	390 ± 103	
	Cervical	244	57
	Lumbar	30	7
	Sacral	1	0
Admission AIS grade	Thoracic	142	33
	A	108	25
	B	48	11
	C	151	35
	D	110	26
AIS conversion from admission to 12 months	A-B	4	0.9
	A-C	4	0.9
	A-D	1	0.2
	B-C	11	2.6
	B-D	4	0.9
	C-D	47	11
	C-E	1	0.2
	D-E	1	0.2
AIS conversion from admission to discharge	A-B	4	0.9
	A-C	4	0.9
	B-C	13	3
	B-D	4	0.9
	C-D	47	11
	D-E	3	0.7

Time periods are ± interquartile range.

SCI, spinal cord injury; AIS, American Spinal Injury Association Impairment Scale.

The remaining 417 patients had their initial blood sample taken at a mean of 31 ± 30 (standard deviation) days post-injury. Blood measures that had been assessed in less than 50% of the patient cohort were excluded. The remaining blood measures included adjusted calcium estimate, alkaline phosphatase, C-reactive protein (CRP), hematocrit, hemoglobin, mean cell hemoglobin, mean cell volume, mononucleocytes, platelets, potassium, red blood count, red blood distance width, and white blood count (WBC). Routine blood analyses were conducted in the Hematology and Biochemistry department located at the Robert Jones and Agnes Hunt Orthopedic Hospital. Hematology analyses were performed on either a Beckman Coulter LH-500 (Beckman Coulter, High Wycombe) or a Sysmex XN-1000 (Sysmex America, IL). Biochemical analyses used VITROS slides (dry multi-layered chemistry slides) in conjunction with the VITROS 5,1 FS Chemistry System (Ortho Clinical Diagnostics, NJ) to measure albumin, ALT, calcium, creatinine, GGT, potassium, magnesium, sodium, total bilirubin, total protein, and urea.

**FIG. 1.**
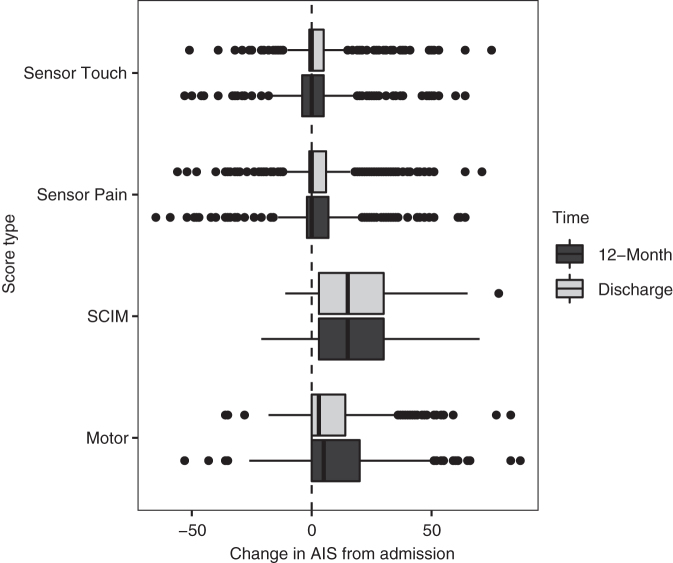
Boxplots of American Spinal Injury Association Impairment Scale (AIS) score change from admission. SCIM, Spinal Cord Independence Measure.

In addition to AIS overall grade, AIS motor, sensory touch, and sensory pin prick scores were recorded at admission, discharge (mean 136 days post-injury ±72), and approximately 12 months post-injury (mean 424 days post-injury ±147). SCIM-III assessments also were recorded at these same time-points.^24^ The SCIM assessment is a disability scale developed to quantify the ability of SCI patients to perform basic activities of independent daily living, including self-care (feeding, bathing, and dressing), respiration and sphincter management, and mobility (Fig. 1).^25,26^

Additional information that may impact neurological recovery and/or the assessed blood measures were included. The incidence of diabetes (types I and II), smoking, and alcohol drinking status were recorded as binary. The neurological level of the injury was recorded as being cervical, thoracic, lumbar, or sacral. Details were recorded as to whether the injury was traumatic, and whether there were any fractures at the injury site. Age at injury in years, gender, and the time between injury and the first blood tests in days also were included. Medications that patients were prescribed also were collected; however, after filtering to drugs at least 50% of patients were given, the remaining drugs were either painkillers or anti-spasm medication. As the inclusion of these drug data would have added a large number of variables to the model and they correlated strongly with initial injury severity, these data were not included in the modeling process.

### Statistical analysis

Data analyses were performed with the statistical programming language R version 3.6.3 (2020-02-29).^27–40^ Missing blood measures were median imputed, then scaled and centered. Less than 21% of the initial and discharge AIS/SCIM scores were missing, whereas 50-60% of the 12-month scores were missing (Supplementary Table S1). These missing AIS grades or scores were imputed with either last observation carried forwards (LOCF) or next observation carried backwards (NOCB) where relevant. LOCF and NOCB were used as it is unusual for AIS or SCIM scores to have decreased over time in SCI patients. These scores typically only either remain largely unchanged, or improve with time.^41^ Therefore, the use of this imputation effectively assumes that in cases of missing score data, the patients' score did not change. This assumption can only worsen model performance, as opposed to giving rise to the overly optimistic models that could be generated by more complex multiple imputation techniques. Additionally, we have been advised that most cases where neurological assessment was missing at admission or discharge is due to a transition from Frankel scoring to AIS. In the case of missing 12-month assessments, this is most commonly due to a given patient not attending their appointment or having received follow up from a different hospital (Supplementary Table S1).

As the number of model features was relatively high compared with the number of observations (45 features and 417 observations), linear regression with elastic net penalization was performed in addition to linear regression without any penalization. Elastic net penalization is a hybrid of ridge regression (whereby the penalty term shrinks predictor effect equally and never to 0), and least absolute shrinkage and selection operator, whereby the penalty term shrinks each predictor differently and allows variables to be removed entirely by shrinking coefficients to 0.^42,43^ Put simply, elastic net reduces the impact of less important model features and can effectively eliminate features entirely, thus performing variable selection during the model building process, as opposed to other methods such as backward variable selection, which are conducted before model building and eliminate features based on co-linearity. Elastic net penalization has been previously found to perform well in models with numerous predictors and in the presence of correlated predictors.^44^

Eight independent models were generated, with and without elastic net penalization, to determine if the features could predict four outcome measures AIS motor, AIS sensor touch, AIS sensor prick, and SCIM, at two time-points: discharge and 12 months post-injury. The data were randomly split 80-20%, whereupon 80% was used for training the model and the remaining 20% was used to test the model's performance. To reduce model overfitting, internal validation was performed by 10-fold cross-validation.^45^

## Results

**FIG. 2.**
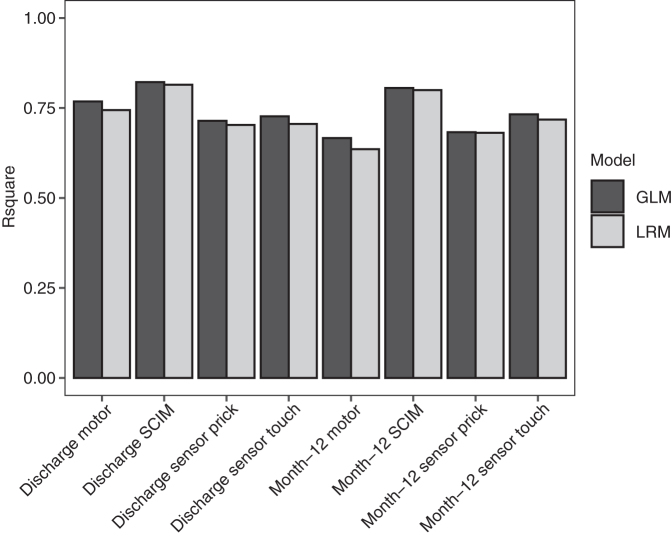
*R*^2^ for models of neurological outcome at discharge and 12 months post-injury. GLM, generalized linear model; LRM, linear regression model; SCIM, Spinal Cord Independence Measure.

**FIG. 3.**
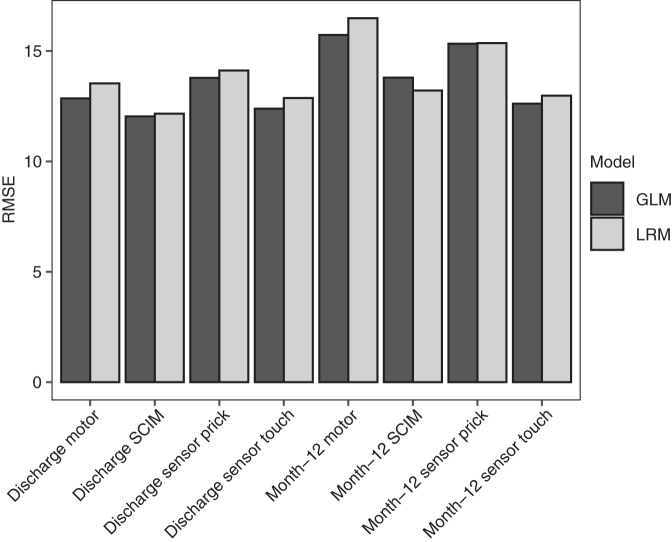
Root mean square error (RMSE) for linear regression models with and without elastic net penalization (GLM and LRM, respectively) of neurological outcome at discharge and 12 months post-injury. GLM, generalized linear model; LRM, linear regression model; SCIM, Spinal Cord Independence Measure.

Multiple regression models of the AIS motor and sensory scores and of SCIM at discharge (mean 136 ± 72 days post-injury) and approximately 12 months post injury (mean 424 ± 147 days post-injury) were built (Supplementary Tables S2, S3, and S4). In addition to standard linear regression models (LRMs), generalized linear models (GLMs) with elastic net penalization also were performed. The modeling techniques performed similarly (GLM *R*^2^ range 0.56-0.79 and root mean square error (RMSE) range 11-18; LRM *R*^2^ range 0.53-0.76 and RMSE range 12-19; Fig. 2 and Fig. 3)

### Model features

With respect to model features, AIS measures of initial neurological function were the most consistently conserved features and the most powerful predictors of outcome measures for the generalized models. Initial SCIM was also included for all the models of outcome, except those relating to discharge sensory prick, touch and 12-month sensory touch. The blood markers, ALT, albumin, alkaline phosphatase, CRP, creatinine, GGT, hematocrit, hemoglobin, mean cell hemoglobin, mean cell volume, monocytes, platelets, potassium, total bilirubin, total protein, urea, and WBC were significant (*p* < 0.05) or included in one or more models (Table 2). 

**Table 2.** Counts of Model Feature Occurrence

Model feature	GLM	LRM
(Intercept)	8	8
Admission AIS grade B	2	2
Admission AIS grade C	6	6
Admission AIS grade D	6	6
Age at injury	2	2
Alanine transaminase (μ/L)	2	0
Albumin (g/L)	1	0
Alkaline phosphatase (μ/L)	1	0
C-reactive protein (mg/L)	1	0
Creatinine (μmol/L)	4	2
Drinking yes	5	1
Fracture	1	1
Gamma GT (μ/L)	1	0
Hematocrit (L/L)	4	0
Hemoglobin (g/L)	5	0
Initial motor	8	6
Initial SCIM	4	2
Initial sensor prick	8	2
Initial sensor touch	5	3
Lumbar injury	2	0
Mean cell Hb (pg)	4	0
Mean cell volume (fL)	6	0
Monocytes (10^*^9/L)	7	0
Neurological level T	1	0
Platelets (10^*^9/L)	1	0
Potassium (mmol/L)	1	0
Sex	2	1
Smoker status known	1	0
Smoker status unknown	0	1
Surgery	1	0
Time to first blood test (days)	0	2
Total bilirubin (μmol/L)	5	3
Total protein (g/L)	1	0
Type 1 diabetes	2	0
Type 2 diabetes	3	1
Urea (mmol/L)	1	1
White blood count (10^*^9/L)	1	0

For unpenalized LRM, statistically significant (*p* < 0.05) features are included. For penalized models (GLM), features that were not penalized to 0 are induced.

GLM, generalized linear model; LRM, linear regression models; AIS, American Spinal Injury Association Impairment Scale; GT, glutamyl transferase; SCIM, Spinal Cord Independence Measure; Hb, hemoglobin.

For the linear regression models, the AIS grade on admission was the only feature that was statistically significant (*p* < 0.05) in all models except 12-month SCIM. The initial measure of the model target—the initial AIS motor score for the models of discharge and 12-month AIS motor for example—also was significant in all models. Other significant features that were not blood measures included diabetes and smoker status, age at injury, time until first blood test from injury, the neurological level of injury, gender, and the presence of fracture at the injury site. With regard to blood measures, urea, monocytes, mean cell hemoglobin, mean cell volume, hematocrit, and hemoglobin were all significant in one or more of the models (Table 2).

### Model performance

**FIG. 4.**
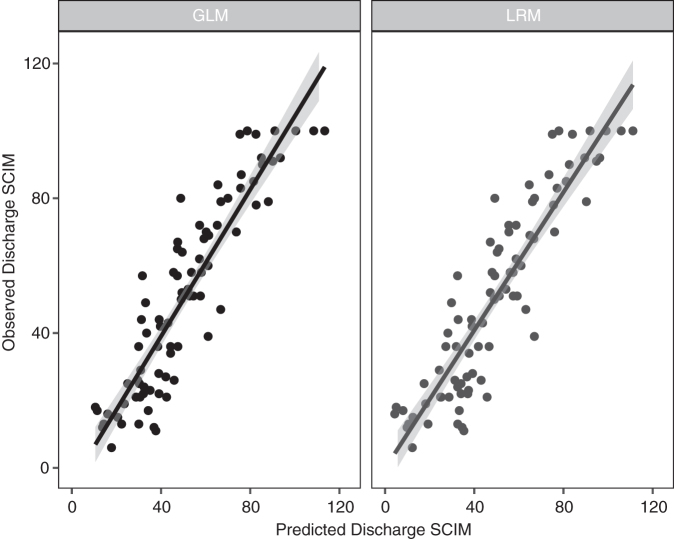
Predicted Spinal Cord Independence Measure (SCIM) score at discharge compared with the observed SCIM scores in the test data.

With respect to model predictions, both modeling techniques performed similarly when predicting against the test data (Figs. 4 and S1 S2 S3 S4 S5 S6 S7). 

## Discussion

Penalized GLM was compared with linear regression in the study due to the sample size. While there has long been a dogma that 10 events per variable is sufficient, more recent studies have argued that there is no rational for this.^46,47^ As there were 417 patients and 45 variables, we also investigated the impact of modeling with and without variable selection in the form of elastic net penalization.

In this study, a standard linear regression model with no variable selection performed very similarly to GLM with elastic net penalization with respect to *R*^2^ and RMSE, although the *R*^2^ of GLM was slightly higher and RMSE slightly lower for all model targets (Fig. 1 and Fig. 2). This suggests that elastic net penalization does not provide a substantial boost to overall model performance at this sample size relative to linear regression. However, there was a difference in the variables each model utilized.

With regard to blood measures in the linear regression models, urea, total bilirubin and creatinine were significant predictors for one or more outcomes. Creatinine was predictive of discharge SCIM and sensor touch. Total bilirubin was predictive motor, sensor prick, and sensor touch at Month 12, suggesting it is predictive of longer-term outcomes. Urea, which is typically used as an indicator of kidney function but may also be altered due to hydration status, was predictive of discharge SCIM in the standard linear regression model, but was predictive of Month 12 sensor touch in the penalized models.

With the exception of time to first blood test from injury, all of the same features were included in the penalized models and the linear regression models, but other related bloods were also included, such as mean cell hemoglobin, mean cell volume, hematocrit, hemoglobin, platelets, and WBC, which are the components of a complete blood count. The complete blood count is likely related to the initial injury severity via blood loss due to bony soft tissue or visceral injury, gastrointestinal bleeding, and/or surgery.^48^ Monocytes were included in all GLM models at both time-points except Month 12 SCIM. Similar to the components of the complete blood count, monocytes levels may be indicative of anemia (if low), but have also been associated with hepatitis and inflammatory diseases (if high).^49,50^ Estimated serum creatinine, based on glomerular filtration rates, are typically used in the evaluation of renal function.^51,52^ SCI patients also have been found to have an increased risk of renal deterioration and are recommended to receive lifelong, regular renal and upper urinary tract examinations after injury.^53,54^ SCI has been found to lead to systemic inflammation which can in turn cause secondary organ complications, including in the liver, kidneys, and lungs, which may explain why these blood measures are useful in predicting outcome.^55–58^

Some studies have found SCI to induce hepatic lipid deposition and inflammation within 3 months of injury in rats, which is symptomatic of non-alcoholic steatohepatitis, the hepatic presentation of metabolic syndrome.^59,60^ Importantly, the blood measures associated with liver function (alanine transaminase, alkaline phosphatase, CRP, GGT, and total bilirubin) highlighted in this study also were found to be significantly predictive of AIS scores in our preliminary study. Two factors—“liver function,” consisting of alanine transaminase, alkaline phosphatase and GGT, and “liver function and inflammation,” consisting of CRP and total bilirubin—added statistically significant value to models of AIS touch and pain scores at 3 months post-injury, and AIS motor and pain scores at 12 months.^23,61,62^ Total bilirubin in particular was included in five of eight penalized models and was significant in three of the non-penalized models. This provides further evidence that liver function is relevant to neurological recovery in SCI.

Interestingly, alanine transaminase, alkaline phosphatase, GGT, and albumin were only retained in the models of SCIM. This could be because these markers indicate liver status, which in turn typically reflects general metabolic health. Therefore, aberrant ALT and GGT values may be a proxy measure of poor metabolic health or systemic inflammation. Diabetes status was also significant in six of the 16 models built in this study, which may also reflect the relevance of general metabolic health in recovery. Metabolic syndrome is also more common in SCI patients than the general population and, SCI patients consequently have an increased risk of diabetes, stroke, and heart disease.^60,63-65^

Serum albumin also has been previously found to be significantly predictive of AIS grade improvement up to 52 weeks.^66^ Platelets and gender also were only retained in models of SCIM. Previous studies contradict this result and have suggested that gender does not significantly correlate with functional neurology or independence.^67,68^ However, it may be that some elements of the SCIM questionnaire are easier for males, such as self-catheterization, and so they are able to obtain slightly higher scores than females, even at a similar level of neurological function (as determined by AIS scores). Interestingly, surgery was only found to be a significant predictor of SCIM at both time-points in the GLM models. This suggests surgery does not have a substantial influence on AIS outcomes. It should be stressed that this hospital favors a conservative approach to care of SCI patients, only choosing to operate in the most extreme cases and so both the rate and type of surgery given to this cohort likely differ from other spinal centers.^69^ Therefore, external validation with data from centers with the more common surgical approach to SCI care is needed to more fully establish the role of surgery in predicting outcomes.^70,71^

Whether the injury was traumatic or not was not retained in any model. Despite the distinct pathophysiology of non-traumatic injuries, this data suggests trauma status is not a strong predictor of AIS motor or sensor score outcomes.^72^ Prior studies have also observed similar functional outcomes between traumatic and non-traumatic injuries.^73^ Further research is needed to establish the role of the liver in SCI, particularly whether the liver is causally implicated in functional recovery, or if it is merely a proxy indicator of systemic inflammation inhibiting healing. Once this association is established, clinicians could consider monitoring the liver function of SCI patients more closely, perhaps attempting to restore/maintain healthy parameters in the interim by minimizing the use of hepatotoxic drugs where possible.

An important limitation of this study is the volume and completeness of the data used in model building. A larger sample size will always lead to a more robust and widely applicable model, and while there was enough to build linear regression models, a larger dataset (> 5000) could allow for robust logistic regression models to predict a change in AIS grade. Further, the data used here contained missing values, and while these were imputed to have minimal effect on model performance, it is still preferable to have a complete dataset. Models of 12-month outcomes were built using discharge and admission scores with the same methodology, and while these models performed better overall, the proportion of missing values at the 12-month time-point, sample size, and more modest difference in average AIS score between discharge and 12 months may cause overfitting, therefore this data was not included. Finally, an independent external validation of these models on separate data, potentially with a cohort with more typical surgical based care, would be desirable, particularly for the GLMs as it is difficult to obtain robust estimates of bias in penalized regression, making standard errors and confidence intervals inappropriate.^74^

## Conclusion

The results from this study suggest that routinely measured blood analytes can provide useful prognostic information for AIS scores and SCIM assessments up to 12 months post-injury, reinforcing the findings of our preliminary study.^23^ Markers of liver function are of particular interest, and rehabilitation clinicians should consider the maintenance of liver health as a priority as it may be relevant to neurologic functional recovery. More research is needed to establish whether or not the relationship between SCI recovery and liver function is causal. Ultimately these finding need to be validated on a larger independent cohort before any firm clinical recommendations can be made.

## Supplementary Material

Supplemental data

Supplemental data

Supplemental data

Supplemental data

Supplemental data

Supplemental data
