# Supplementary material for: Routinely Measured Hematological Markers Can Help to Predict American Spinal Injury Association Impairment Scale Scores after Spinal Cord Injury
Source: J Neurotrauma. 2021 Jan 15;38(3):301–8. doi: 10.1089/neu.2020.7144 (PMC7826437; doi:10.1089/neu.2020.7144)
Supplement: Supplemental data [file Supp_Tables1-3.pdf]

Supplementary Data

Supplementary Table S1. Missing AIS and SCIM Scores (Out of 431 Total Patients)

	Total number of missing values	*Percent missing*
Initial sensor prick	3	1
Initial sensor touch	3	1
Initial motor	4	1
Discharge SCIM	37	9
Initial SCIM	44	11
Discharge motor	73	18
Discharge sensor prick	87	21
Discharge sensor touch	87	21
Month 12 SCIM	231	55
Month 12 motor	252	60
Month 12 sensor touch	255	61
Month 12 sensor prick	256	61

AIS, American Spinal Injury Association Impairment Scale; SCIM, Spinal Cord Independence Measure.

Supplementary Table S2. Linear Regression Model Coefficients with Elastic Net Penalization

Model	*Variable*	*Coefficients*
Discharge motor	(Intercept)	14.4
	Hemoglobin (g/L)	0.56
	Mean cell Hb (pg)	0.343
	Mean cell volume (fL)	0.297
	Monocytes (10*9/L)	0.714
	Admission ASIA C	8.12
	Admission ASIA D	7.27
	Alcohol-drinking status	0.665
Discharge sensor prick	Initial motor	0.681
	Initial sensor prick	0.0847
	Initial sensor touch	0.00759
	Initial SCIM	0.0483
	(Intercept)	16.7
	Creatinine (μmol/L)	0.215
	Hemoglobin (g/L)	0.98
	Monocytes (10*9/L)	0.936
	Total bilirubin (μmol/L)	0.961
	Type 2 diabetes	0.13
Discharge sensor touch	Admission ASIA C	5.97
	Admission ASIA D	1.51
	Initial motor	0.165
	Initial sensor prick	0.564
	Initial sensor touch	0.13
	(Intercept)	21.3
	Creatinine (μmol/L)	1.05
	Hematocrit (L/L)	1.55
	Mean cell volume (fL)	0.225
	Monocytes (10*9/L)	1.11
	Total bilirubin (μmol/L)	0.758
Discharge SCIM	Type 2 diabetes	1.96
	Admission ASIA B	2.85
	Admission ASIA C	10.3
	Admission ASIA D	5.24
	Lumbar injury	0.489
	Alcohol-drinking status	0.046
	Initial motor	0.064
	Initial sensor prick	0.0308
	Initial sensor touch	0.654
	(Intercept)	24.1
	Alanine transaminase (μ/L)	0.743
	Albumin (g/L)	0.000168
	Alkaline phosphatase (μ/L)	0.732
	Creatinine (μmol/L)	1.48
	Gamma GT (μ/L)	0.189
	Mean cell volume (fL)	0.899
	Monocytes (10*9/L)	0.0577
	Platelets (10*9/L)	0.249
	Total protein (g/L)	0.775
	White blood count (10*9/L)	0.697
	Type 1 diabetes	4.86
Month 12 motor	Neurological level T	0.984
	Sex	1.97
	Alcohol-drinking status	1.88
	Fracture	0.697
	Surgery	1.27
	Initial motor	0.263
	Initial sensor prick	0.0828

(*continued*)

Supplementary Table S2. (Continued)

Model	*Variable*	*Coefficients*
Month 12 sensor prick	Initial SCIM	0.577
	(Intercept)	20.5
	Creatinine (μmol/L)	0.016
	C-reactive protein (mg/L)	0.868
	Hematocrit (L/L)	0.00315
	Hemoglobin (g/L)	1.64
	Mean cell Hb (pg)	0.0101
	Mean cell volume (fL)	0.442
	Monocytes (10*9/L)	0.852
	Potassium (mmol/L)	0.404
Month 12 sensor touch	Total bilirubin (μmol/L)	0.386
	Type 1 diabetes	3.37
	Admission ASIA B	0.277
	Admission ASIA C	9.01
	Admission ASIA D	9.39
	Smoking yes	0.699
	Alcohol-drinking status	1.75
	Initial motor	0.576
	Initial sensor prick	0.126
	Initial SCIM	0.027
	(Intercept)	14.3
	Hematocrit (L/L)	0.425
Month 12 SCIM	Hemoglobin (g/L)	0.74
	Mean cell Hb (pg)	0.209
	Monocytes (10*9/L)	0.986
	Total bilirubin (μmol/L)	1.12
	Admission ASIA C	6.03
	Admission ASIA D	1.44
	Lumbar injury	1.45
	Age at injury (median years)	0.0507
	Initial motor	0.193
	Initial sensor prick	0.423
	Initial sensor touch	0.229
	(Intercept)	15.5
	Hematocrit (L/L)	0.779
	Hemoglobin (g/L)	0.777
	Mean cell Hb (pg)	0.00573
	Mean cell volume (fL)	0.07
	Monocytes (10*9/L)	1.17
	Total bilirubin (μmol/L)	0.672
	Urea (mmol/L)	0.162
	Type 2 diabetes	1.87
	Admission ASIA C	8.83
	Admission ASIA D	2.44
	Age at injury (median years)	0.0249
	Alcohol-drinking status	0.65
	Initial motor	0.0837
	Initial sensor prick	0.0852
	Initial sensor touch	0.632
	(Intercept)	23.9
	Alanine transaminase (μ/L)	0.168
	Mean cell volume (fL)	0.136
	Sex	1.47
	Initial motor	0.221
	Initial sensor prick	0.0909
	Initial SCIM	0.591

Hb, hemoglobin; AISA, American Spinal Injury Association; SCIM, Spinal Cord Independence Measure; GT, glutamyl transferase.

Supplementary Table S3. Final Elastic Net Model Parameters

Model target	*alpha*	*lambda*
Discharge motor	1	0.679
Discharge sensor prick	1	0.738
Discharge sensor touch	0.6	0.718
Discharge SCIM	0.6	0.632
Month 12 motor	1	0.636
Month 12 sensor prick	0.4	1.67
Month 12 sensor touch	1	0.722
Month 12 SCIM	1	1.47

Alpha is a value between 0 and 1, where 0 is pure ridge regression, 1 is pure LASSO and values between are a mixture of both. Lambda is the shrinkage factor applied to model coefficients.

SCIM, Spinal Cord Independence Measure.
