# Supplementary material for: Routinely Measured Hematological Markers Can Help to Predict American Spinal Injury Association Impairment Scale Scores after Spinal Cord Injury
Source: J Neurotrauma. 2021 Jan 15;38(3):301–8. doi: 10.1089/neu.2020.7144 (PMC7826437; doi:10.1089/neu.2020.7144)
Supplement: Supplemental data [file Supp_Table4.pdf]

Supplementary Table S4. Linear Regression Model Coefficients without Elastic Net Penalization

| *Model* | *Variable* | *Estimate* | *Standard Error* | *t value* | *Pr(>\|t\|)* |
|---|---|---|---|---|---|
| Discharge motor | (Intercept) | 19.6 | 5.02 | 3.91 | 0.000117 |
| | Admission ASIA C | 12.4 | 2.54 | 4.89 | 1.64e-06 |
| | Admission ASIA D | 11.2 | 3.41 | 3.3 | 0.0011 |
| Discharge sensor prick | Initial motor | 0.705 | 0.0635 | 11.1 | 4.15e-24 |
| | (Intercept) | 14.8 | 5.21 | 2.85 | 0.00472 |
| | Total bilirubin ($\mu$mol/L) | 1.4 | 0.596 | 2.36 | 0.0192 |
| | Admission ASIA C | 9.91 | 2.64 | 3.76 | 0.000209 |
| | Initial motor | 0.21 | 0.0659 | 3.19 | 0.00158 |
| Discharge sensor touch | Initial sensor prick | 0.555 | 0.0611 | 9.09 | 1.59e-17 |
| | (Intercept) | 22.4 | 4.54 | 4.94 | 1.32e-06 |
| | Creatinine ($\mu$mol/L) | 1.84 | 0.935 | 1.97 | 0.0496 |
| | Admission ASIA B | 6.96 | 2.46 | 2.83 | 0.00492 |
| | Admission ASIA C | 14.5 | 2.3 | 6.29 | 1.17e-09 |
| | Admission ASIA D | 9.96 | 3.09 | 3.23 | 0.00139 |
| | Initial sensor touch | 0.664 | 0.0537 | 12.4 | 1.67e-28 |
| | (Intercept) | 24.1 | 5.79 | 4.17 | 4.06e-05 |
| | Creatinine ($\mu$mol/L) | 2.96 | 1.19 | 2.48 | 0.0138 |
| Discharge SCIM | Urea (mmol/L) | -2.72 | 1.25 | -2.18 | 0.03 |
| | Type 2 diabetes | -6.68 | 2.85 | -2.34 | 0.0198 |
| | Admission ASIA B | -7.26 | 3.14 | -2.32 | 0.0213 |
| | Admission ASIA D | -10.6 | 3.94 | -2.68 | 0.0078 |
| | Age at injury (median years) | -0.208 | 0.0616 | -3.38 | 0.000827 |
| | Time to first blood test (days) | -0.109 | 0.0393 | -2.78 | 0.00586 |
| | Smoking unknown | -5.18 | 2.36 | -2.2 | 0.0289 |
| | Initial motor | 0.301 | 0.0733 | 4.11 | 5.19e-05 |
| | Initial SCIM | 0.591 | 0.0605 | 9.78 | 1.02e-19 |
| | (Intercept) | 25.7 | 6.1 | 4.2 | 3.51e-05 |
| Month 12 motor | Admission ASIA C | 14.6 | 3.09 | 4.72 | 3.67e-06 |
| | Admission ASIA D | 15.6 | 4.15 | 3.76 | 0.000206 |
| | Initial motor | 0.571 | 0.0772 | 7.39 | 1.52e-12 |
| | (Intercept) | 13.1 | 5.59 | 2.35 | 0.0194 |
| | Total bilirubin ($\mu$mol/L) | 1.57 | 0.64 | 2.45 | 0.0149 |
| | Admission ASIA C | 11.3 | 2.83 | 3.99 | 8.25e-05 |
| Month 12 sensor prick | Admission ASIA D | 7.75 | 3.8 | 2.04 | 0.0422 |
| | Fracture | -4.39 | 2.19 | -2 | 0.0461 |
| | Initial motor | 0.209 | 0.0708 | 2.95 | 0.00344 |
| | Initial sensor prick | 0.434 | 0.0656 | 6.62 | 1.75e-10 |
| | Initial sensor touch | 0.173 | 0.0661 | 2.62 | 0.00922 |
| | (Intercept) | 17.6 | 5.33 | 3.3 | 0.00111 |
| | Total Bilirubin ($\mu$mol/L) | 1.37 | 0.611 | 2.25 | 0.0255 |
| | Admission ASIA C | 13.8 | 2.7 | 5.1 | 6.06e-07 |
| Month 12 sensor touch | Admission ASIA D | 8.18 | 3.62 | 2.26 | 0.0248 |
| | Drinker status | 3.57 | 1.66 | 2.15 | 0.0321 |
| | Initial sensor touch | 0.618 | 0.0631 | 9.79 | 9.19e-20 |
| | (Intercept) | 20.9 | 6.47 | 3.23 | 0.00139 |
| | Sex | 4.73 | 2.2 | 2.15 | 0.032 |
| | Age at injury (median years) | -0.226 | 0.0687 | -3.29 | 0.00111 |
| | Time to first blood test (days) | -0.103 | 0.0439 | -2.36 | 0.0191 |
| | Initial motor | 0.275 | 0.0819 | 3.36 | 0.000896 |
| Month 12 SCIM | Initial SCIM | 0.595 | 0.0675 | 8.81 | 1.17e-16 |

AISA, American Spinal Injury Association; SCIM, Spinal Cord Independence Measure.
