# Supplementary material for: Routinely Measured Hematological Markers Can Help to Predict American Spinal Injury Association Impairment Scale Scores after Spinal Cord Injury
Source: J Neurotrauma. 2021 Jan 15;38(3):301–8. doi: 10.1089/neu.2020.7144 (PMC7826437; doi:10.1089/neu.2020.7144)
Supplement: Supplemental data [file Supp_Figs1-2.pdf]

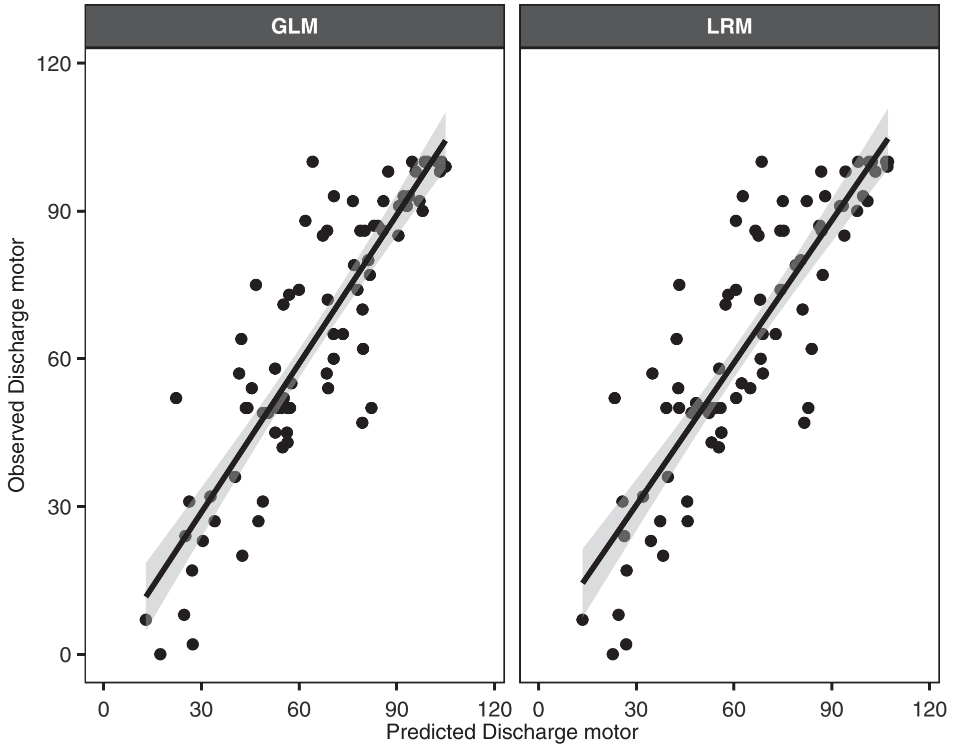

SUPPLEMENTARY FIG. S1. Predicted motor score at discharge compared to the observed motor scores in the test data.

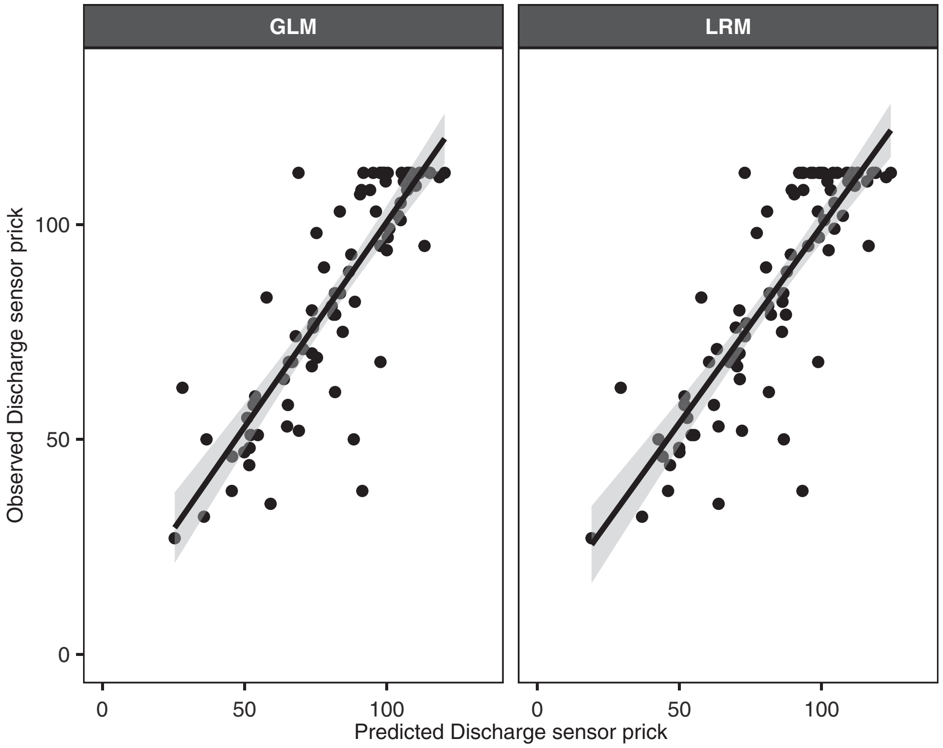

SUPPLEMENTARY FIG. S2. Predicted sensor prick score at discharge compared to the observed sensor prick scores in the test data.
