# Supplementary material for: Routinely Measured Hematological Markers Can Help to Predict American Spinal Injury Association Impairment Scale Scores after Spinal Cord Injury
Source: J Neurotrauma. 2021 Jan 15;38(3):301–8. doi: 10.1089/neu.2020.7144 (PMC7826437; doi:10.1089/neu.2020.7144)
Supplement: Supplemental data [file Supp_Figs5-6.pdf]

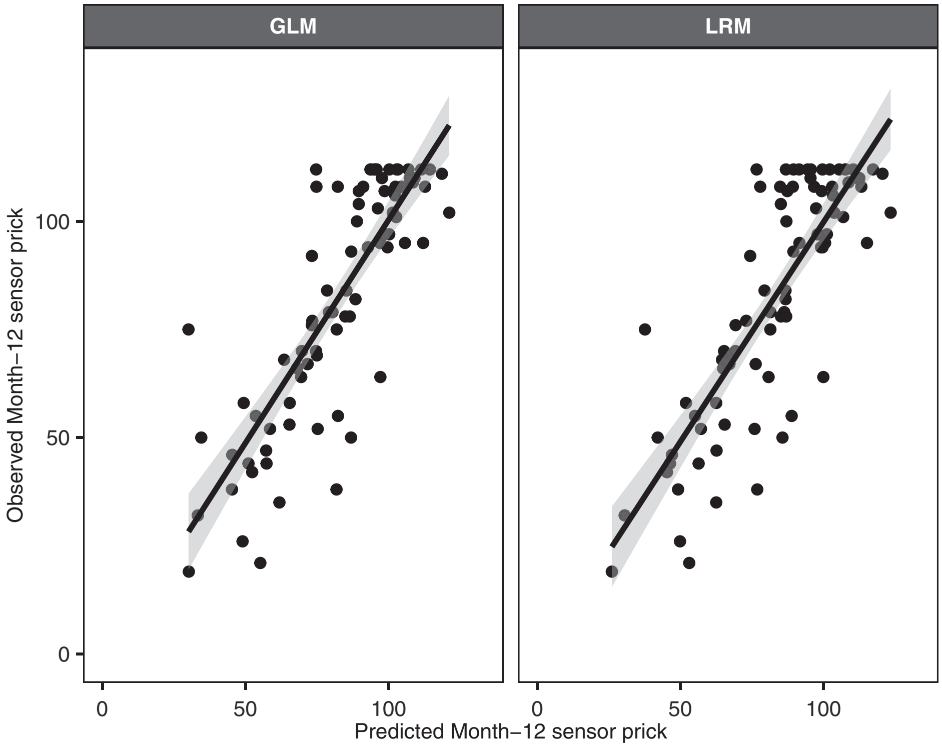

SUPPLEMENTARY FIG. S5. Predicted sensor prick score at month-12 compared to the observed month-12 sensor prick in the test data.

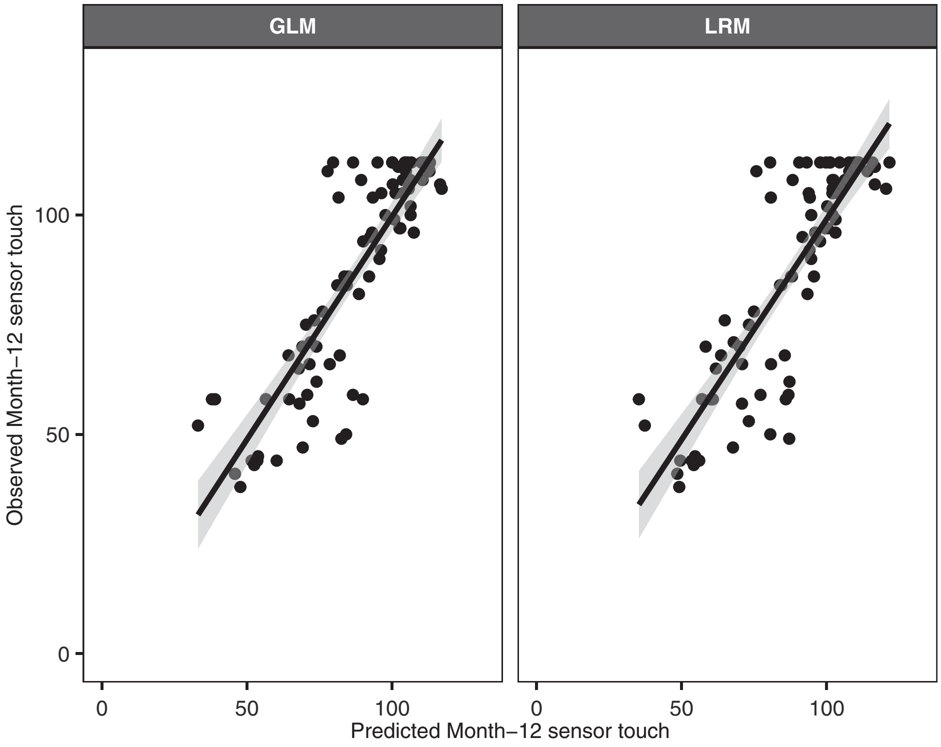

SUPPLEMENTARY FIG. S6. Predicted sensor touch score at month-12 compared to the observed sensor touch in the test data.
